# Factors Associated with Fruit and Vegetable Intake Among Women of Reproductive Age in Flint, Michigan: A Cross-Sectional Analysis †

**DOI:** 10.3390/healthcare13192399

**Published:** 2025-09-23

**Authors:** Gayle Shipp, Diana K. Haggerty, Amy Saxe-Custack, Jenny LaChance, Nicole Jones

**Affiliations:** 1 Charles Stewart Mott Department of Public Health, College of Human Medicine, Michigan State University, Flint, MI 48502, USA; hagger39@msu.edu (D.K.H.); saxeamym@msu.edu (A.S.-C.); lachan14@msu.edu (J.L.); warnerni@msu.edu (N.J.); 2 Michigan State University–Hurley Children’s Hospital Pediatric Public Health Initiative, College of Human Medicine, Michigan State University, Flint, MI 48502, USA; 3 Department of Pediatrics and Human Development, College of Human Medicine, Michigan State University, Flint, MI 48502, USA

**Keywords:** nutritional status, psychological stress, perceived health and wellbeing

## Abstract

**Background/Objectives:** There is a paucity of research evaluating factors associated with healthy eating behaviors in reproductive-age, non-pregnant women. This study aims to examine the association between sociodemographic, perceived health, and environmental factors with fruit and vegetable (F&V) consumption and adherence to dietary recommendations among women of reproductive age who lived, worked, or attended school in Flint, Michigan, during the Flint water crisis (April 2014–October 2015). **Methods:** This cross-sectional study used data collected from enrollment surveys completed by participants in the Flint Registry (December 2019–2021). Inclusion criteria were females aged 18–55 having complete data on sociodemographic indicator, general health perception, and dietary assessment data (n = 1239). Pearson chi-square measures of association were used to compare frequencies within groups between sociodemographic characteristics, perceived health and wellbeing (PHW), F&V quality/selection, adherence to recommendations, and total F&V intake. **Results:** Few participants met recommendations for fruits (22.8%) and vegetables (20.3%). Approximately 50.5% reported having access to a wide variety of F&Vs in their neighborhood and perceived F&V quality to be high. Factors significantly associated with meeting F&V recommendations included education, income, homeownership status, and PHW (*p* < 0.05). **Conclusions:** Associations between environmental and socioeconomic factors and daily F&V intake among women of reproductive age offer important considerations for designing interventions to improve this demographic’s nutritional status. Results indicate low adherence to recommended F&V intake but perceived access and quality as high in this population. Targeted interventions addressing key components of socioeconomic barriers, perceived access and quality, and perceived wellbeing are needed to improve F&V intake.

## 1. Introduction

Regular intake of high-nutrient fruits and vegetables (F&Vs) reduces risk of diet-related chronic health conditions, including diabetes, heart disease and certain types of cancer [1]. The Dietary Guidelines for Americans, 2020–2025, recommend daily consumption of 1.5–2 cups of fruits and 2–3 cups of vegetables as part of an overall healthy diet [2]. During the reproductive period, a “healthy diet” is defined by higher consumption of seafood, poultry, whole grains, and F&Vs [3]. This diet is crucial for the health and maintenance of the reproductive system and helps reduce the risk of chronic health conditions in women [4]. Despite evidence linking health outcomes to nutrition, adherence to dietary guidelines remains low: most US adults fail to meet the recommended daily intake (≥1.5 cups of fruit and ≥2 cups of vegetables) [2]. Moreover, increasing evidence indicates women are disproportionately affected by chronic conditions associated with poor dietary behaviors [5].

While the benefits of adequate F&V consumption are well-established, several barriers to achieving dietary recommendations exist (e.g., limited access to/availability of fresh F&Vs). There is a growing need for tailored interventions to increase F&V intake to reduce age, sex, racial/ethnic, and income disparities among US adults [2]. To date, few US studies have examined factors associated with F&V consumption and adherence to dietary recommendations among women of reproductive age [6]. Maintaining a healthy diet in this population is critical for several reasons, including supporting fertility and fecundity, promoting healthy perinatal outcomes, and reducing the risk of chronic health conditions [7,8]. A recent study noted that education was associated with a “prudent” style diet (i.e., nutrient-dense) in this population but noted the paucity of research on this topic in the target population [6]. Creating nutrition interventions first requires identifying and understanding factors that may be associated with eating behaviors. Therefore, we aimed to characterize the associations of sociodemographic, health and environmental factors with F&V consumption and adherence to dietary recommendations in a unique population of reproductive-aged women affected by the Flint water crisis.

## 2. Materials and Methods

We used data collected at enrollment from participants enrolled in the Flint Registry, a public health surveillance system for people exposed to the Flint water crisis [9]. Participants provided written informed consent before participation. The Michigan State University Institutional Review Board approved secondary analyses of data (STUDY00005777). We included participants whose surveys were completed between 1 December 2019 and 31 December 2021. Recruitment and data collection for the Flint Registry are described elsewhere [9,10]. Briefly, the Flint Registry was open to anyone who lived, worked, or attended school or day care in the city of Flint or who was *in utero* and whose mother met eligibility criteria [10]. Descriptive analyses were limited to adult non-pregnant or -lactating female (self-identified) participants of reproductive age (18–55) having complete data on variables of interest, including sociodemographic indicators, general health perceptions, and dietary assessments (n = 1239).

Based on known associations from the literature, self-reported health and financial indicators included age, education, marital status, employment status, combined family income in the past 12 months, home ownership (own, rent, or other arrangement not specified), and living standard adjustment from loss of income (see table for categories used). Living standard adjustment from loss of income was measured by asking participants how long they could maintain their current standard of living if they lost all sources of income. Environmental indicators included perceived availability of high-quality fresh F&Vs and of a large selection of F&Vs in neighborhood. Using a perceived health and wellbeing (PHW) subjective tool, perceptions of general health, physical health, mental health, and quality of life were measured on a 5-point Likert scale [11]. Reported values were dichotomized into ‘Fair/Poor’ versus ‘Good/Very Good/Excellent’ for analysis. We categorized the Likert scale to be consistent with reporting for similar health and quality of life questions used by the Michigan Behavioral Risk Factor Surveillance System [12]. Body mass index (BMI) was calculated using self-reported weight and height.

The F&V screener in Eating at America’s Table Study [13] was used to collect F&V intake, calculated based on participant-reported number of servings and average serving size of foods. Reported intake was totaled/scored and summarized as cup-equivalents per day based upon food type (fruit and/or vegetable). Total intake (cups/day) for F&V was categorized in ordinal categories (<one cup, one cup but <two cups, two cups but <three cups, three or more cups) and binary categories were determined by recommended consumption for adults (≥one and a half cups of fruits, ≥two cups of vegetables).

The Pearson chi-square test was applied to compare frequencies within groups between sociodemographic and social characteristics, PHW, F&V quality/selection, adherence to recommendations based on 2020–2025 Dietary Guidelines for Americans [14], and total intake of F&Vs (Stata command tabulate with chi-square test [15]). We allowed denominators to vary when characteristic data were missing. Stata version 14.0 was used. *p* values < 0.05 were considered statistically significant.

## 3. Results

Among study participants, 47.9% (n = 593) were between 18 and 34 years of age. Most reported having some college education (42.1%, n = 519) or higher (21.4%, n = 264) (Supplementary Table S1). Overall, 22.8% (n = 283) met current U.S. Dietary Guidelines 2020–2025 for fruits and 20.3% (n = 252) did so for vegetables. The total daily F&V intake showed 30.4% (n = 376) of participants consumed less than one cup per day, 28.3% (n = 351) ate between one and two cups/day, 15.3% (n = 190) ate between two and three cups/day, and 26.0% (n = 322) consumed three or more cups/day. Most participants responded “good,” “very good,” or “excellent” to general health questions: 820 (66.7%); quality of life: 901 (73.7%); physical health: 746 (61.2%); and mental health: 722 (58.8%).

There were few significant differences in daily adherence to F&V recommendations observed in relation to socioeconomic factors (Table 1). Meeting recommendations for fruit intake varied according to family income and home ownership status, whereas meeting recommendations for vegetables varied according to educational attainment and living standard adjustment from loss of income. For example, 36.3% (n = 325) of women who reported not meeting daily fruit intake recommendations reported that their annual family income was less than USD 12,000 and 50.9% (n = 137) of women who reported meeting recommendations reported their annual family income as less than USD 12,000. Of the women who did not meet the daily recommended vegetable intake, 19.8% (n = 194) had a Bachelor’s Degree or higher, whereas 27.5% (n = 69) of women who met recommendations for daily vegetable intake had a Bachelor’s Degree or higher.

Neighborhood access variables were associated with meeting recommendations for fruit intake only (Table 2). Among women who did not report meeting daily fruit intake recommendations, 45.3% (n = 412) indicated they strongly agreed or agreed with the statement that the F&Vs available in their neighborhood were high quality, whereas 53.5% (n = 146) of those who did meet daily fruit recommendations reported they strongly agreed or agreed with the statement. Results were similar for the question that asked if a large selection of F&Vs was available in their neighborhoods.

Meeting recommendations for fruit intake was associated with perceived physical health only (Table 3). Among women who did not meet fruit intake recommendations, 40.4% (n = 380) reported their physical health as poor or fair, whereas 33.5% (n = 93) of women who met fruit intake recommendations reported their physical health as poor or fair. Meeting recommendations for vegetable intake was associated with perceived quality of life, physical health, and mental health, of which mental health demonstrated the largest differences between those who did and did not meet vegetable recommendations. Among those who did not meet vegetable intake recommendations, 43.5% (n = 426) reported their mental health as poor or fair, whereas 31.9% (n = 79) of those who did meet recommendations reported poor or fair mental health.

Fruit and vegetable intake, calculated as cups per day, was associated with combined family income and living standard adjustment from loss of income but was not significantly associated with other sociodemographic factors (Table 4). Among those who reported eating less than one cup of fruits and vegetables per day, 36.1% (n = 125) reported an annual family income of USD 11,999 or less, whereas 47.1% (n = 144) of participants who ate three or more cups of fruits and vegetables per day reported an annual income of USD 11,999 or less. However, standard of living adjustment from loss of income followed a different pattern, with 54.1% (n = 139) of those who reported eating less than one cup of fruits and vegetables daily being able to maintain their standard of living for less than one month compared to 41.9% (n = 90) of those who ate three or more cups per day being able to maintain their standard of living for less than one month.

Fruit and vegetable intake was associated with neighborhood availability of high quality and large selections of fruits and vegetables in a dose-like manner (Table 5). For example, strong agreement or agreement with the statement “The fresh fruits and vegetables in my neighborhood are of high quality” increased from 40.3% (n = 146) among those who reported eating less than one cup of fruits and vegetable per day to 46.1% (n = 150) among those who ate one to less than two cups per day, to 51.6% (n = 96) among those who reported eating two to less than three cups per day, and to 53.7% (n = 166) among those who reported eating 3 or more cups per day.

PHW was also associated with F&V intake in a dose response manner (Table 6), particularly for physical and mental health. For example, the prevalence of poor or fair physical health decreased as F&V daily intake increased, with 44.9% (n = 166) of those who ate less than one cup per day reporting poor or fair physical health, 41.5% (n = 144) of those who ate one to less than two cups per day reporting poor or fair physical health, 33.7% (n = 62) of those who ate two to less than three cups per day reporting poor or fair physical health, and 31.8% (n = 101) of those who ate three or more cups per day reporting poor or fair physical health.

## 4. Discussion

Among reproductive-aged women participating in the Flint Registry, many fail to meet current F&V recommendations. Only 20.3% reported eating the recommended daily amount of vegetables, and 22.8% reported eating the recommended daily amount of fruits. Sociodemographic variations in F&V intake in this study are consistent with the existing literature, which has noted similar differences based on education, employment, home ownership, and combined family income [16]. Neighborhood availability and perceived quality of F&Vs were associated with F&V intake, which suggests that improving access to and quality of F&Vs may support increased consumption. In 2019, it was estimated that only 12.3% of adults met recommendations related to fruit intake and 10% met recommendations for vegetable intake [2], and a cross-sectional study of prenatal patients in Flint found 30.6% met fruit recommendations, while only 8.3% met vegetable recommendations in 2022–2023 [17]. Although the frequency of meeting recommended F&V intake was higher in our sample compared to national data, it may have been influenced by the expansion of programs that supported nutrition for people exposed to the Flint water crisis [18].

In this study, combined F&V intake was significantly associated with all four domains of PHW. A recent systematic review evaluated perceived stress and diet quality and indicated various studies had mixed results. Authors reported an inverse association between stress and diet, noting that few studies focused on women of reproductive age [19]. Additionally, few studies have investigated the relationship between PHW and F&V consumption. This indicates a need for research to explore and clarify associations and potential synergistic effects between these factors, especially among a demographic that may be motivated to change behaviors when planning a family [20].

Between April 2014 and October 2015, the city of Flint experienced municipal water supply contamination that garnered national attention and became known as the Flint water crisis [21,22]. An estimated 140,000 people were exposed to the contamination [22]. At the same time, researchers in Flint identified neighborhood-level disparities in the availability of healthy food options for Flint residents [23]. Critical to the Flint water crisis response was the expansion of food distribution sites that focused on delivering foods high in calcium, vitamin C, and other nutrients that help mitigate the effects of lead exposure [18,24]. However, quality of food at community distribution sites can vary according to the ways in which food is distributed, and evidence suggests that food distribution sites that allow recipients to select their foods have higher quality foods available than sites that provide pre-bagged food packages [25]. In response to the COVID-19 pandemic, many food sites in Flint relied on a pre-bagged distribution system [24]. Though our study does not evaluate the use of food distribution centers by our participants, our finding that participants who strongly agreed that the selection and quality of the fruits and vegetables in their neighborhood was high more frequently ate three or more cups of fruits and vegetables per day compared to those who reported they disagreed points to the importance of the food environment when making these selections and highlights the importance of making fresh fruits and vegetables available where people live. An estimated 20,413 women of reproductive age resided in the city of Flint during the years of data collection [26], and identifying the factors associated with F&V intake, such as access, can inform food access initiatives that serve this population.

Identifying sociodemographic, behavior, and health factors associated with diet behaviors is crucial for developing effective interventions that improve diet quality among a demographic that may be motivated to optimize diet for the purposes of family planning. Bedrick et al. found that women with a college degree were more likely to follow a “prudent” or nutrient-dense diet including vegetables, fruits, nuts, and olive oil. Comparatively, our study had similar findings, where a higher frequency of women met fruit and vegetable recommendations than those who had less educational attainment [6]. Other measures of socioeconomic position, perceptions of quality and selection of fruits and vegetables in neighborhoods, and duration of time one could sustain their current standard of living if all sources of income were lost were also potential determinants of F&V intake in our study. This suggests that intake of F&Vs is not dictated by a single socioeconomic construct. Additional studies that address sociodemographic patterns and their associations with F&V intake are essential for better understanding how these characteristics vary across populations.

### Limitations

This study is a cross-sectional evaluation of associations between F&V intake with participant characteristics and cannot evaluate causation. Potential confounding was not accounted for in the bi-variable associations, and residual confounding cannot be ruled out. The sample is a subset of Flint Registry participants who completed the food intake screener and may not represent the full population of reproductive-age women living in Flint. The Flint Registry relies on self-reported measures due to lack of access to medical records, which may result in recall bias for variables such as body mass index and the food intake screener. The recall duration may result in measurement error around amounts and types of foods eaten, and so we elected to report fruit and vegetable intake results as categorical to help reduce error.

## 5. Conclusions

This study highlights the associations between F&V adherence and daily intake among women of reproductive age in the target area. Our study identified PHW and neighborhood food environment as novel predictors of F&V intake in women of reproductive age. Given the novel associations we identified, future research should consider multivariable modeling to assess independent effects, explore underlying mechanisms, and begin designing interventions and policy initiatives aimed at improving neighborhood food environments, health perceptions, and nutritional status of women in this demographic.

## Figures and Tables

**Table 1:** Pearson chi-square measures of associations between sociodemographics and adherence to recommendations among women of reproductive age in Flint, Michigan (December 2019–2021).

Characteristics	Recommended Fruit Intake ^a^ %		Recommended Vegetable Intake ^a^ %	
	Did Not Meet Recommendation	Met Recommendation	Did Not Meet Recommendation	Met Recommendation
**Age, n**	956	283	987	252
18–34 years	47.5	49.5	47.1	51.2
35–55 years	52.5	50.5	52.9	48.8
**Educational Level, n**	951	281	981	251
Some High School or less	8.8	12.1	9.6 *	9.6 *
High School Graduate or GED	25.9	30.6	27.0 *	26.7 *
Some college, Associate’s Degree, or Technical or vocational training	43.0	39.2	43.6 *	36.2 *
Bachelor’s Degree or higher	22.3	18.1	19.8 *	27.5 *
**Marital Status, n**	944	280	975	249
Divorced/Widowed/Separated	17.5	17.1	17.7	16.1
Married/Live together	24.9	20.7	24.4	22.1
Never Married	57.6	62.1	57.9	61.8
**Employment Status, n**	925	273	957	241
Employed	62.2	57.1	61.2	60.1
Not employed, not looking for work	16.9	16.9	16.4	18.7
Not employed, looking for work	20.9	26.0	22.4	21.2
**Combined Family Income—Past 12 Months, n**	894	269	926	237
≤USD 11,999	36.3 ***	50.9 ***	39.7	39.7
USD 12,000–USD 49,000	47.0 ***	37.9 ***	45.9	40.9
≥USD 50,000	16.7 ***	11.2 ***	14.4	19.4
**Home Ownership, n**	935	277	967	245
Own	35.8 ***	22.0 ***	32.3	34.3
Rent	52.6 ***	64.3 ***	56.3	51.4
Other arrangement	11.6 ***	13.7 ***	11.4	14.3
**Body Mass Index (BMI), n**	899	264	933	230
Underweight (<18.5)	1.6	4.2	2.0	2.6
Normal Weight (18.5–24.9)	17.5	18.9	17.2	20.4
Overweight (25.0–29.9)	21.2	19.7	21.2	19.6
Obese (>30.0)	59.7	57.2	59.6	57.4
**Living Standard Adjustment from Loss of Income, n**	685	181	692	174
<1 month	46.4	42.5	46.5 *	41.9 *
1–2 months	31.7	27.1	32.0 *	25.9 *
3–6 months	10.7	17.7	10.7 *	17.8 *
7–12 months	11.2	12.7	10.8 *	14.4 *

^a^ Adult dietary recommendations are ≥1.5–2 cup equivalents of fruits and ≥2–3 cup equivalents of vegetables daily. *p* value is for all differences/overall comparison. *** *p* < 0.001, ** *p* < 0.01, * *p* < 0.05.

**Table 2:** Pearson chi-square measures of associations between neighborhood fruit and vegetable access and adherence to recommendations among women of reproductive age in Flint, Michigan (December 2019–2021).

Characteristics	Recommended Fruit Intake ^a^ %		Recommended Vegetable Intake ^a^ %	
	Did Not Meet Recommendation	Met Recommendation	Did Not Meet Recommendation	Met Recommendation
**Fresh F&Vs Available Are of High Quality, n**	909	273	939	243
Strongly agree or agree	45.3 *	53.5 *	46.1	51.4
Neither agree or disagree	35.1 *	26.4 *	33.8	30.5
Strongly disagree or disagree	19.6 *	20.1 *	20.1	18.1
**Large Selection of F&Vs Are Available in Neighborhood, n**	940	281	972	249
Strongly agree or agree	48.0 ***	58.4 ***	49.9	52.2
Neither agree or disagree	28.2 ***	18.1 ***	26.4	23.7
Strongly disagree or disagree	23.8 ***	23.5 ***	23.7	24.1

^a^ Adult dietary recommendations are ≥1.5–2 cup equivalents of fruits and ≥2–3 cup equivalents of vegetables daily. *p* value is for all differences/overall comparison. *** *p* < 0.001, ** *p* < 0.01, * *p* < 0.05.

**Table 3:** Pearson chi-square measures of associations between perceived health and wellbeing and adherence to recommendations among women of reproductive age in Flint, Michigan (December 2019–2021).

Characteristics	Recommended Fruit Intake ^a^ %		Recommended Vegetable Intake ^a^ %	
	Did Not Meet Recommendation	Met Recommendation	Did Not Meet Recommendation	Met Recommendation
**Perceived General Health, n**	951	280	981	250
Excellent, Very Good, or Good	66.1	68.2	65.9	69.2
Fair or Poor	33.9	31.8	34.1	30.8
**Perceived Quality of Life, n**	944	279	972	249
Excellent, Very Good, or Good	72.7	77.4	72.5 *	78.7 *
Fair or Poor	27.3	22.6	27.5 *	21.3 *
**Perceived Physical Health, n**	941	278	971	248
Excellent, Very Good, or Good	59.6 *	66.5 *	59.4 *	68.1 *
Fair or Poor	40.4 *	33.5 *	40.6 *	31.9 *
**Perceived Mental Health, n**	948	279	979	248
Excellent, Very Good, or Good	57.4	63.8	56.5 ***	68.1 ***
Fair or Poor	42.6	36.2	43.5 ***	31.9 ***

^a^ Adult dietary recommendations are ≥1.5–2 cup equivalents of fruits and ≥2–3 cup equivalents of vegetables daily. *p* value is for all differences/overall comparison. *** *p* < 0.001, ** *p* < 0.01, * *p* < 0.05.

**Table 4:** Pearson chi-square measures of associations between sociodemographics and combined fruit and vegetable intake (cups) among women of reproductive age in Flint, Michigan (December 2019–2021).

Characteristics	Fruit and Vegetable Intake %			
	<1 Cup	≥1 Cup but <2 Cups	≥2 Cups but <3 Cups	≥3 Cups
**Age, n**	376	351	190	322
18–34 years	45.5	47.2	48.4	51.2
35–55 years	54.5	52.7	51.6	48.8
**Educational Level, n**	374	348	190	320
Some High School or less	7.8	10.1	10.5	10.6
High School Graduate or GED	27.5	27.0	23.7	28.1
Some college, Associate’s Degree, or Technical or vocational training	46.5	42.2	39.0	38.8
Bachelor’s Degree or higher	18.1	20.7	26.8	22.5
**Marital Status, n**	372	345	188	319
Divorced/Widowed/Separated	19.1	17.4	17.0	15.7
Married/Live together	26.3	24.1	26.6	19.4
Never Married	54.6	58.5	56.4	64.9
**Employment Status, n**	358	343	185	312
Employed	59.5	60.1	68.7	59.3
Not employed, not looking for work	17.9	17.5	13.5	17.0
Not employed, looking for work	22.6	22.4	17.8	23.7
**Combined Family Income—Past 12 Months, n**	346	327	184	306
≤USD 11,999	36.1 **	37.9 **	37.5 **	47.1 **
USD 12,000–USD 49,000	51.5 **	46.2 **	42.9 **	37.2 **
≥USD 50,000	12.4 **	15.9 **	19.6 **	15.7 **
**Home Ownership, n**	370	343	185	314
Own	32.7	35.0	36.2	28.0
Rent	57.0	53.9	48.1	58.9
Other arrangement	10.3	11.1	15.7	13.1
**Body Mass Index (BMI), n**	356	333	174	300
Underweight (<18.5)	2.2	1.2	2.9	2.7
Normal Weight (18.5–24.9)	16.9	18.0	14.9	20.3
Overweight (25.0–29.9)	20.2	22.2	17.2	22.3
Obese (>30.0)	60.7	58.6	65.0	54.7
**Living Standard Adjustment from Loss of Income, n**	257	248	146	215
<1 month	54.1 **	44.8 **	37.7 **	41.9 **
1–2 months	26.5 **	35.5 **	35.6 **	27.0 **
3–6 months	10.1 **	10.0 **	10.9 **	17.7 **
7–12 months	9.3 **	9.7 **	15.8 **	13.4 **

^a^ Adult dietary recommendations are ≥1.5–2 cup equivalents of fruits and ≥2–3 cup equivalents of vegetables daily. *p* value is for all differences/overall comparison. *** *p* < 0.001, ** *p* < 0.01, * *p* < 0.05.

**Table 5:** Pearson chi-square measures of associations between neighborhood fruit and vegetable access and combined fruit and vegetable intake (cups) among women of reproductive age in Flint, Michigan (December 2019–2021).

Characteristics	Fruit and Vegetable Intake %			
	<1 Cup	≥1 Cup but <2 Cups	≥2 Cups but <3 Cups	≥3 Cups
**Fresh F&Vs Available Are of High Quality, n**	362	325	186	309
Strongly agree or agree	40.3 **	46.1 **	51.6 **	53.7 **
Neither agree or disagree	38.4 **	36.0 **	27.4 **	27.2 **
Strongly disagree or disagree	21.3 **	17.9 **	21.0 **	19.1 **
**Large Selection of F&Vs Are Available in Neighborhood, n**	371	345	187	318
Strongly agree or agree	42.0 **	51.6 **	55.1 **	56.0 **
Neither agree or disagree	30.2 **	27.8 **	20.9 **	21.7 **
Strongly disagree or disagree	27.8 **	20.6 **	24.0 **	22.3 **

^a^ Adult dietary recommendations are ≥1.5–2 cup equivalents of fruits and ≥2–3 cup equivalents of vegetables daily. *p* value is for all differences/overall comparison. *** *p* < 0.001, ** *p* < 0.01, * *p* < 0.05.

**Table 6:** Pearson chi-square measures of associations between perceived health and wellbeing and combined fruit and vegetable intake (cups) among women of reproductive age in Flint, Michigan (December 2019–2021).

Characteristics	Fruit and Vegetable Intake %			
	<1 Cup	≥1 Cup but <2 Cups	≥2 Cups but <3 Cups	≥3 Cups
**Perceived General Health, n**	374	351	187	319
Excellent, Very Good, or Good	62.6 *	64.7 *	72.2 *	70.2 *
Fair or Poor	37.4 *	35.3 *	27.8 *	29.8 *
**Perceived Quality of Life, n**	372	348	185	318
Excellent, Very Good, or Good	68.8 **	71.5 **	81.1 **	77.7 **
Fair or Poor	31.2 **	28.5 **	18.9 **	22.3 **
**Perceived Physical Health, n**	370	350	185	318
Excellent, Very Good, or Good	55.1 ***	58.5 ***	66.3 ***	68.2 ***
Fair or Poor	44.9 ***	41.5 ***	33.7 ***	31.8 ***
**Perceived Mental Health, n**	374	350	185	318
Excellent, Very Good, or Good	50.5 ***	58.0 ***	64.3 ***	66.4 ***
Fair or Poor	49.5 ***	42.0 ***	35.7 ***	33.6 ***

^a^ *p* value is for all differences/overall comparison. *** *p* < 0.001, ** *p* < 0.01, * *p* < 0.05.

## Data Availability

Data available on request due to restrictions.

## Supplementary Materials

The following supporting information can be downloaded at: https://www.mdpi.com/article/10.3390/healthcare13192399/s1, Table S1: Descriptive characteristics of Flint Registry women of reproductive age in Flint, Michigan (December 2019–2021).
