# Supplementary material for: Factors Associated with Fruit and Vegetable Intake Among Women of Reproductive Age in Flint, Michigan: A Cross-Sectional Analysis
Source: Healthcare (Basel). 2025 Sep 23;13(19):2399. doi: 10.3390/healthcare13192399 (PMC12523652; doi:10.3390/healthcare13192399)
Supplement: Supplementary file 1 [file healthcare-13-02399-s001.zip › healthcare-3825450-supplementary.pdf]

Supplementary Material

Table S1. Descriptive characteristics of Flint Registry women of reproductive age in Flint, Michigan (December 2019–2021).

Characteristics	%
**Age, n**	1239
18-34 years	47.9
35-55 years	52.1
**Educational Level, n**	1232
Some High School or less	9.6
High School Graduate or GED	26.9
Some college, Associate's Degree, or Technical or vocational training	42.1
Bachelor's Degree or higher	21.4
Missing, n	7
**Marital Status, n**	1224
Divorced/Widowed/Separated	17.4
Married/Live together	23.9
Never Married	59.7
Missing, n	15
**Employment Status, n**	1198
Employed	61
Not employed, not looking for work	16.9
Not employed, looking for work	22.1
Missing, n	41
**Combined Family Income—Past 12 Months, n**	1163
≤$11,999	39.7
$12,000- $49,000	44.9
≥$50,000	15.4
Missing, n	76
**Home Ownership, n**	1212
Own	32.7
Rent	55.3
Other arrangement	12.1
Missing, n	27
**Body Mass Index (BMI), n**	1163
Underweight (<18.5)	2.2
Normal Weight (18.5-24.9)	17.8
Overweight (25.0-29.9)	20.9
Obese (>30.0)	59.7
Missing, n	76
**Living Standard Adjustment from Loss of Income, n**	866
<1 month	45.6
1-2 months	30.7
3-6 months	12.1
7-12 months	11.6
Missing, n	373

**Fresh F&V Available Are of High Quality, n**	1182
Strongly agree or agree	47.2
Neither agree or disagree	33.1
Strongly disagree or disagree	19.7
Missing, n	57
**Large Selection of F&V Are Available in Neighborhood, n**	1221
Strongly agree or agree	50.4
Neither agree or disagree	25.9
Strongly disagree or disagree	23.7
Missing, n	18
**Perceived General Health, n**	1231
Excellent, Very Good, or Good	66.7
Fair or Poor	33.3
Missing, n	8
**Perceived Quality of Life, n**	1223
Excellent, Very Good, or Good	73.7
Fair or Poor	26.3
Missing, n	16
**Perceived Physical Health, n**	1219
Excellent, Very Good, or Good	61.2
Fair or Poor	38.8
Missing, n	20
**Perceived Mental Health, n**	1227
Excellent, Very Good, or Good	58.8
Fair or Poor	41.2
Missing, n	12
**Recommended Fruit Intake, n**	1239
Did not meet recommendation	77.2
Met recommendations	22.8
**Recommended Vegetable Intake, n**	1239
Did not meet recommendation	79.7
Met recommendations	20.3
**Fruit and Vegetable Intake, n**	1239
<1 cup	30.4
≥1 cup but <2 cups	28.3
≥2 cups but <3 cups	15.3
≥3 cups	26
